# Serological Evidence of Zika Virus Circulation in Burkina Faso

**DOI:** 10.3390/pathogens11070741

**Published:** 2022-06-29

**Authors:** Bachirou Tinto, Didier Patindé Alexandre Kaboré, Dramane Kania, Thérèse Samdapawindé Kagoné, Alice Kiba-Koumaré, Laura Pinceloup, Guillaume Thaurignac, Philippe Van de Perre, Roch Kounbobr Dabire, Thierry Baldet, Serafin Guitierrez, Patricia Gil, Ahidjo Ayouba, Sara Salinas, Yannick Simonin

**Affiliations:** 1 Pathogenesis and Control of Chronic and Emerging Infections, INSERM, University of Montpellier, 34000 Montpellier, France; laura.pinceloup-sosa@etu.umontpellier.fr (L.P.); p-van_de_perre@chu-montpellier.fr (P.V.d.P.); 2 Centre MURAZ, Institut National de Santé Publique (INSP), Bobo-Dioulasso BP 390, Burkina Faso; draka3703@yahoo.fr (D.K.); tskagone@gmail.com (T.S.K.); 3 Institut de Recherche en Sciences de la Santé (IRSS), Bobo-Dioulasso BP 390, Burkina Faso; kapda0123@gmail.com (D.P.A.K.); dabireroch@gmail.com (R.K.D.); 4 Centre National de Transfusion Sanguine, Ouagadougou BP 5372, Burkina Faso; alice_kiba@yahoo.fr; 5 Recherches Translationnelles sur le VIH et Maladies Infectieuses, Université de Montpellier, IRD, Inserm, 34000 Montpellier, France; guillaume.thaurignac@ird.fr (G.T.); ahidjo.ayouba@ird.fr (A.A.); 6 ASTRE Research Unit, CIRAD, INRAe, Montpellier University, 34000 Montpellier, France; thierry.baldet@cirad.fr (T.B.); serafin.gutierrez@cirad.fr (S.G.); patricia.gil@cirad.fr (P.G.)

**Keywords:** Zika virus, dengue virus, flavivirus, arbovirus, seroprevalence, Burkina Faso

## Abstract

Zika virus (ZIKV) and dengue virus (DENV) are two closely related members of the *Flaviviridae* family, both transmitted by mosquitoes of the genus *Aedes*, and are among the arboviruses most at risk to human health. Burkina Faso has been facing an upsurge in DENV outbreaks since 2013. Unlike DENV, there is no serological evidence of ZIKV circulation in humans in Burkina Faso. The main objective of our study was to determine the seroprevalence of ZIKV and DENV in blood donors in Burkina Faso. A total of 501 donor samples collected in the two major cities of the country in 2020 were first tested by a competitive enzyme-linked immunosorbent assay to detect flavivirus antibodies. Positive sera were then tested using Luminex to detect ZIKV and DENV antibodies and virus-specific microneutralization tests against ZIKV were performed. The ZIKV seroprevalence was 22.75% in the donor samples and we found seropositivity for all DENV-serotypes ranging from 19.56% for DENV-1 to 48.86% for DENV-2. Molecular analyses performed on samples from febrile patients and *Aedes aegypti* mosquitoes between 2019 and 2021 were negative. Our study showed the important circulation of ZIKV and DENV detected by serology although molecular evidence of the circulation of ZIKV could not be demonstrated. It is essential to strengthen existing arbovirus surveillance in Burkina Faso and more broadly in West Africa by focusing on fevers of unknown origin and integrating vector surveillance to assess the extent of ZIKV circulation and identify the circulating strain. Further studies are needed to better understand the epidemiology of this virus in order to define appropriate prevention and response methods.

## 1. Introduction

Zika (ZIKV) and dengue (DENV) viruses are arthropod-borne viruses (arboviruses) belonging to the *Flaviviridae* family of the *Flavivirus* genus. These viruses are transmitted to humans through the bite of infected mosquitoes from the *Aedes* genus, mainly *Aedes egypti* and *Aedes albopictus* [1,2]. There are also cases of non-vectorial transmission by blood transfusion, accidental exposure to biological fluids among healthcare personnel or nosocomial transmission, mother-to-child transmission (vertical transmission), and sexual transmission, in particular with ZIKV [1,2]. Importantly, the clinical diagnosis of these viruses is difficult in areas endemic to malaria because the symptoms may appear similar [3].

ZIKV is endemic in all tropical regions of the world: Africa, the Americas, and Southeast Asia [4]. The virus was first identified in 1947 in a rhesus monkey in the Zika forest in Uganda and the first human case was reported in 1954 in Nigeria [5]. Only 14 human cases were reported before 2007 [6]. Similar to many arboviruses, the majority of ZIKV-infected patients are asymptomatic [7]. The clinical picture of ZIKV infection was correctly established after the epidemic in Yap in 2007, with fever, rash, arthralgia, and conjunctivitis as the most common symptoms [8]. The same clinical picture was observed during the outbreaks in French Polynesia and Brazil, in 2013 and 2015, respectively [9,10]. During the later epidemics, numerous cases of neurological complications such as Guillain-Barre and neurodevelopmental deficits, (e.g., microcephaly) in children born to infected mothers as well as other neurological syndromes such as meningitis or meningoencephalitis have been observed [11].

DENV is endemic in more than 100 countries, mostly in urban and semi-urban areas, in Africa, Asia, and America. These last two continents are the most affected. The global incidence of dengue has grown dramatically with about half of the world’s population now at risk. Circulation of the virus in West Africa was first reported in Nigeria in the 1960s [12]. Dengue fever is the most common arboviral disease in the world and is due to four different serotypes: DENV-1, DENV-2, DENV-3, and DENV-4 [13]. Infection with one of the serotypes does not confer long-term cross-protection [2]. The disease is asymptomatic in the majority of cases. However, some patients experience symptoms such as fever, myalgia, arthralgia, anorexia, retro-orbital pain, nausea and vomiting, sore throat, headache, and rash. Neurological and bleeding complications may occur in some cases [14]. There is no specific treatment for dengue/severe dengue. Early detection of disease progression associated with severe dengue, and access to proper medical care lowers fatality rates of severe dengue to below 1%. Studies have shown that sequential infection with different DENV serotypes increases the risk of complications that can lead to death [15]. This phenomenon called antibody-dependent enhancement (ADE) is also observed in patients who have been exposed to ZIKV and subsequently contracted dengue [16].

In Burkina Faso, there has been an upsurge in dengue fever epidemics since 2013 [2]. All four DENV serotypes have already been identified in the country since the first suspected case was reported in 1925 [2]. Unlike DENV, there is no molecular and serological evidence of ZIKV circulation in humans in this country. The objective of our study was to determine the seroprevalence in Burkina Faso of ZIKV and DENV in blood donors and to carry out a molecular screening of ZIKV on samples of febrile patients and mosquitoes (*Aedes aegypti*).

## 2. Results

### 2.1. Serological Screening for ZIKV and DENV in Blood Donor

To assess the circulation of ZIKV and the different DENV serotypes in Burkina Faso, we analyzed blood donor samples from two regional blood transfusion centers for the year 2020, including 114 (22.75%) women (median age: 28 years; interquartile range (IQR): 24–35.75 years) and 387 (77.25%) men (median age: 28 years; IQR 24–35 years). From those 501 serum samples screened by competitive enzyme-linked immunoabsorbent assay (cELISA) to detect prior flavivirus infection, we identified antibodies against flaviviruses in 400 samples (79.84%, 95% CI: 76.10–83.12). These cELISA-positive samples were then tested with Luminex-based serological assay using the NS1 antigens of ZIKV and for the four DENV serotypes (DENV-1, DENV-2, DENV-3, and DENV-4). We found 229 samples positive for ZIKV (representing 45.70% of the total samples, CI 95%: 41.39–50.08), 98 positives for DENV-1 (19.56%, 95% CI: 16.32–23.26), 280 positives for DENV-2 (48.86%, 95% CI: 44.79–51.13), 204 DENV-3 positive (40.71%, 95% CI: 36.50–45.07) and 199 DENV-4 positive (39.72%, 95% CI: 35.53–44.06) (Table 1). Ninety-four (18.76%) people carried antibodies against the four DENV serotypes. Since ZIKV is not described as circulating in Burkina Faso, unlike dengue virus, to confirm the presence of anti-ZIKV antibodies in blood donor samples, all ZIKV Luminex-positive samples were tested by microneutralization tests (MNT). We found 114 positives (22.75%, 95% CI: 19.29–26.62) with high neutralizing antibody titer (Table 2).

We did not find a significant association between the sex of blood donors and ZIKV seroprevalence while there was a statistically significant difference between origin and age and ZIKV seroprevalence. Blood donors from Ouagadougou city were the most affected and ZIKV seroprevalence increased with age (Table 3).

### 2.2. Molecular Screening for ZIKV and DENV in Samples from Febrile Patients and Aedes aegypti Mosquitoes

With RT-qPCR, we analyzed ZIKV RNA in samples from febrile patients collected in 2019 as part of the national surveillance of dengue and yellow fever in Burkina Faso. About 26% of samples collected in Burkina Faso in 2019 were positive for DENV PCR. We, therefore, selected 340 samples among the negative DENV samples that we analyzed by RT-PCR for ZIKV. No ZIKV-positive samples were detected in this specific cohort. Additionally, a total of 1356 *Aedes aegypti* no-blood-engorged females were collected in the Hauts-Bassins and southwest region of Burkina Faso, in both rural and urban areas, in 2019 (147 females), 2020 (863 females), and 2021 (346 females). We did not identify the presence of ZIKV or DENV in mosquitoes. It should be noted that the national arbovirus surveillance program reported a few sporadic cases of DENV in 2020 and 2021 without noticing major outbreaks.

## 3. Discussion

DENV has become endemic in Burkina Faso with recurrent outbreaks since 2013 [2]. After the dengue epidemics that the country has experienced, there have been no studies to estimate the level of immunity of the population according to the different serotypes. To be fully immune to new dengue infections, it is necessary to have antibodies against all four serotypes. In our study we identified all four DENV serotypes in the blood donor cohort with a predominance of serotype 2 (48.86%); serotype 1 being the least represented (19.56%). Our results are consistent with those obtained in previous studies that reported the presence of the four serotypes, with a predominance of serotype 2, in previous epidemics in the country [17,18,19]. In our study, only 94 people had antibodies against all four serotypes, representing 18.4% of the cohort, which means that the vast majority of people are likely to be infected with different dengue serotypes against which they do not have effective antibodies. For example, an introduction of serotype 1, for which the Burkinabe population has the least antibodies, could lead to new epidemics and potentially severe clinical cases as sequential infections are known to increase the risk of severe dengue [20]. Therefore, monitoring serotypes would be important to anticipate future epidemics in the country. Other studies carried out in the general population in neighboring countries have identified DENV serotypes 2 and 3 in Côte d’Ivoire [21], and DENV serotype 2 in Ghana and Mali [22,23]. All four DENV serotypes have also been identified in Nigeria and Senegal [2,24,25].

Unlike DENV, the circulation of ZIKV has never been clearly established in Burkina Faso despite the surveillance of arboviruses implemented since 2016 [26]. A serological survey of the general population carried out from 1963 to 1964 showed serological evidence of the circulation of the ZIKV in the present-day Republic of Haute-Volta (now Burkina Faso) [27]. Our study is the first to be carried out since the major epidemics of ZIKV the world experienced in 2007, 2013, and 2015 [8,9]. We found a high seroprevalence (22.6%) of ZIKV among blood donors in Burkina Faso. This percentage is close to the 21.9% and 22.7% seroprevalence obtained in Senegal in 2007 and 2011/2012, respectively [28]. Our results are higher than in Mali, a country bordering Burkina Faso, which detected 12% of positives between 2013 and 2016 [29]. We found statistically significant associations between ZIKV seroprevalence and the location of blood donors. Donors from Ouagadougou had a higher seroprevalence (27.73%) compared to those from Bobo-Dioulasso (17.55%). Ouagadougou has a higher population density than Bobo-Dioulasso and also has problems with access to water and sanitation. These factors could increase the risk of vector proliferation and therefore arbovirus transmission. The ZIKV seroprevalence increased with age with a peak in the 35 to 44 age group with a seroprevalence of 32.63%. In Burkina Faso, this age group represents the most active part of the population and therefore spends more time outdoors compared to the younger and older age groups and are likely to be more exposed to mosquito bites. Our results are comparable to those of Diarra et al. who found in a study carried out in Mali that ZIKV seroprevalence increased with age [29]. We did not observe any statistically significant difference between seroprevalence and sex. Men and women seem to be exposed to the same degree as previously reported in Mali [29].

Despite a high seroprevalence, we did not obtain positive results for ZIKV in the RT-qPCR performed on the human fever cohort or mosquito samples. This result implies that either the virus may have been in circulation at low levels during the study period, causing mainly asymptomatic cases within the population, or that the virus may have been transmitted in previous years. Indeed the detection of arbovirus RNA is difficult and rare outside of epidemic periods. Added to this is the short viremia observed during infection with arbovirus including ZIKV [30]. Furthermore, in the literature, we have not encountered any cases of microcephaly in newborns or Guillain-Barre reported in Burkina Faso, even though these two neurological symptoms are typical of complications due to infection with ZIKV. However, these neurological disorders are relatively rare outcomes and African and Asian lineages may have differential virulence [31]. Moreover, it is not clear if the African lineage of ZIKV has the capacity to cause microcephaly or not. In addition, health care systems in some regions are limited and one cannot exclude un-reported cases. It is important to note that RT-PCR testing for DENV in *Aedes aegypti* mosquitoes was negative despite the existence of molecular evidence of DENV circulation in humans in Burkina Faso [18,32]. This result could be due to the low number of mosquitoes tested. In general, the detection of arboviruses in natural mosquito populations is not very sensitive unless mosquito collections are carried out during a major circulation of the virus or during an epidemic [33]. As a reminder, surveillance of arboviruses was initiated in Burkina Faso in 2016, some ten years after the emergence of the first major ZIKV epidemics in Yap and French Polynesia. It is possible that human cases of ZIKV may have gone unnoticed in Burkina Faso or in Africa in general due to the low diagnostic and surveillance capacity of most African countries [34]. Studies showed differential transmission efficiency of the African strain of ZIKV compared to the Asian strain by *Aedes Aegypti* [35,36].

Given that prior exposure to ZIKV could potentially cause an ADE effect in the event of a subsequent dengue virus infection, the circulation of ZIKV could increase the number of severe cases in future dengue fever epidemics in Burkina Faso. The circulation of different dengue serotypes could also be a factor favoring the occurrence of severe forms of the disease [37].

It should be noted that we observed differences between Luminex and MNT tests. This makes sense because the NS1 protein, used for Luminex, is an internal protein, which is not the target of neutralizing antibodies. Furthermore, even for surface proteins, not all specific antibodies are neutralizing. It is therefore logical that we observed higher results with an NS1-based test compared to a test detecting neutralizing antibodies.

Our study has shown a high level of DENV and ZIKV seroprevalence in Burkina Faso although molecular evidence of the circulation of ZIKV could not be demonstrated. It is therefore essential to strengthen the existing arbovirus surveillance by focusing on fevers of unknown origin and by integrating vector surveillance. Our results also show the importance of setting up a good program for the prevention and expanded management of the main arboviruses circulating in Burkina Faso. A retrospective study of newborn cohorts covering the last 10 years would be interesting to identify whether there have been undocumented cases of congenital malformations such as microcephaly that could be attributable to ZIKV.

## 4. Materials and Methods

### 4.1. Samples

#### 4.1.1. Blood Donors

The blood donor samples included in our study came from regional blood transfusion centers in the cities of Ouagadougou and Bobo-Dioulasso. A total of 501 serum samples (256 in Ouagadougou and 245 in Bobo Dioulasso) were collected from June to July 2020. At least 600 µL of serum were taken from each participant and sent to the National Reference Laboratory for Viral Hemorrhagic Fevers at the Centre MURAZ Center in Bobo-Dioulasso to be stored at −80 °C before the serological tests.

#### 4.1.2. Febrile Patients

Three hundred and forty fever samples collected as part of arbovirus surveillance in Burkina Faso in 2019 and stored at −80 °C in the biobank at the Centre MURAZ were included in our study. In the present study, we selected 340 samples that had already been tested with dengue fever RT-PCR and were negative. These samples were then screened with ZIKV RT-PCR in our study.

#### 4.1.3. Mosquito Sampling

Mosquito collection was carried out in August, September 2019; June, July, and October 2020, and May, June 2021. Mosquitoes were collected in several areas from the Hauts-Bassins and southwest region of Burkina Faso. In the Hauts-Bassins region, the sampling was carried out in seven localities: Bobo-Dioulasso, Banakeledaga, Sourkoudougou, Badara, Vallée du Kou 3 (VK3), Nasso and Dinderesso. In the southwest region, sampling was carried out in six sites: Diébougou, Gaoua, Bapla, Tiankoura, Banlo and Bouroum-bouroum.

Sampling was carried out over two successive days in each locality. Two methods were used: the BG-Sentinel traps and the Prokopack aspiration. The various specimens (living) were identified morphologically to the species level using the identification keys [38,39,40]. Non-blood-engorged females of *Aedes aegypti* were sorted and stored at −80 °C for subsequent analyses.

Mosquitoes were pooled in groups of 6 to 37 individuals per site and collection date. Pools were homogenized in 500 µL ice-cold 1X-PBS (Phosphate Buffered Saline) buffer with two ice-cold steel bearing balls (3 mm diameter, LOUDET) using a TissueLyser II (Qiagen). After homogenization and clarification, total RNA was extracted from homogenate supernatants with the NucleoMagVet kit (Macherey Nagel) using a Kingfisher Flex (Thermo Scientific).

### 4.2. Competitive Enzyme-Linked Immunosorbent Assay

All blood donor samples were screened using a competitive ELISA test: the ID Screen^®^ West Nile Competition Multi-species from Innovative Diagnostics (Grabel, France). This test was originally developed for the detection of antibodies directed against the envelope protein pr-E of the West Nile virus but it is found to give cross reactions with other flaviviruses including the ZIKV and DENV and can therefore be used for the screening of these viruses [41]. The test was performed according to the manufacturer’s protocol.

### 4.3. Luminex

All cELISA-positive samples were screened in Luminex using the NS1 antigens of ZIKV and DENV (DENV-1, DENV-2, DENV-3, and DENV-4) according to the procedures described by Raulino et al. [42].

Recombinant NS1 proteins for ZIKV and for the four DENV serotypes were coupled to Luminex beads to detect Immunoglobulin G (IgG) directed against these antigens. The samples, diluted to 1/200th, are placed in the presence of the beads and incubated overnight at 4 °C. The fluorescence intensities of the antigen-antibody reactions are then read with the Bioplex 200 device. A blank is used to measure only the fluorescence intensity of the beads and remove this “background noise” from the other results obtained. The NS1 antigen used for ZIKV has a sensitivity of 100% and a specificity of 98.48%; for DENV-1, a sensitivity of 91.3% and a specificity of 98.48%; for DENV-2, a sensitivity of 100% and a specificity of 96.97%; for DENV-3, a sensitivity of 100% and a specificity of 96.97%, for DENV-1, a sensitivity of 82.61% and a specificity of 98.48%.

### 4.4. Seroneutralization Assays

Viral microneutralization tests (MNT) were performed on cELISA-positive sera to confirm ZIKV infection. The sera were serially diluted in duplicate in 50 µL of Dulbecco’s Modified Eagle Medium (DMEM) of ThermoFisher supplemented by heat-inactivated fetal bovine serum 2% and hepes 40X in a 96 plate, then with a dilution factor of 3 starting with 1/5th to 1/3645th. ZIKV suspension (ArB41644 ZIKV of African lineage) at 107.14 tissue culture infectious dose 50 (TCID50) was then added to each well. After incubation for 90 min at 37 °C with 5% CO_2_, we added 100 µL of DMEM 2% containing 2000 Vero cells E6 per well. The plates were incubated at 37 °C with 5% CO_2_ for 7 days. Antibody titers were calculated by doing the reciprocal of the last dilution at which there are no cytopathic effects.

### 4.5. RT-PCR ZIKV and DENV

Nucleic acids from fever samples were extracted using Qiagen RNA extraction kit (QIAamp Viral RNA Mini Kit, Qiagen, Hilden, Germany). Amplification was performed with the QuantStudio^®^ Real-Time PCR from ThermoFisher Scientific (Waltham, Massachusetts, USA) according to the procedures described by Liu et al. [43]. For mosquitoes, total RNA extraction was performed using the Biomek-FX machine (Beckman-Coulter) and the Nucleospin RNA virus extraction kit (Macherey-Nagel), following the manufacturer’s instructions. Control of the quantity and quality of the RNA was measured by spectrophotometry (Nanodrop, Thermo Fisher Scientific) and by capillary electrophoresis (Bioanalyser, Agilent Technologies). The detection of ZIKV and PAN-DENV by real-time RT-PCR was carried out following the protocol described previously by Liu et al. and Gray et al. [43,44]. We use the following primers and probes:

ZIKV: 

Forward: CGCAGGATCATAGGTGATGAAG

Reverse: CCTGACAACACTAAAATTGGTGC

Probe: VIC-ACAGCACTCCAGGTGTAGACCCTTC-BHQ1

DENV:

Forward: GGATAGACCAGAGATCCTGCTGT

Reverse R1: CATTCCATTTTCTGGCGTTC

Reverse R2: CAATCCATCTTGCGGCGCTC

Probe: FAM-CAGCATCATTCCAGGCACAG-TAMRA

### 4.6. Statistical Analysis

The seroprevalence of ZIKV and DENV was calculated by dividing the number of positive samples by the total number of samples tested, using two-sided exact binomial 95% confidence intervals (95%CI). The correlation between seroprevalence of ZIKV and independent variables such as origin, sex, and age were analyzed using a Pearson chi-square test and/or Fisher’s exact test and odds ratio.

## Figures and Tables

**Table 1:** Luminex and MNT results in blood donor samples tested positive for flaviviruses by Elisa. 2021. *N* = 501.

	Luminex Positive *N* (%)					MNT ZIKV Positive *N* (%)
	DENV1 NS1 Ab (%)	DENV2 NS1 Ab (%)	DENV3 NS1 Ab (%)	DENV4 NS1 Ab (%)	ZIKV NS1 Ab (%)	
Total	98 (19.56)	280 (55.88)	204 (40.71)	199 (39.77)	229 (45.39)	114 (22.75)
Origin						
Ouagadougou	63 (24.60)	164 (64.06)	126 (49.21)	122 (47.65)	127 (49.60)	71 (27.73)
Bobo-Dioulasso	35 (14.28)	116 (47.34)	78 (31.83)	77 (31.42)	102 (41.63)	43 (17.55)
Gender						
Male	69 (17.82)	223 (57.62)	158 (40.82)	159 (41.08)	187 (48.32)	90 (23.25)
Female	29 (25.43)	57 (50.0)	46 (40.34)	40 (35.08)	42 (36.84)	24 (21.05)
Age						
18–24	28 (16.47)	81 (47.64)	53 (31.17)	56 (32.94)	63 (37.05)	27 (15.88)
25–34	37 (18.78)	106 (53.80)	79 (40.10)	70 (35.53)	92 (46.70)	45 (22.84)
35–44	25 (26.31)	67 (70.52)	55 (57.89)	55 (57.89)	54 (56.84)	31 (32.63)
45–59	8 (20.51)	26 (66.66)	17 (43.58)	18 (46.15)	20 (51.28)	11 (28.20)

DENV: Dengue virus; ZIKV: Zika virus; NS: non-structural protein; Ab: antibody; MNT: microneutralization test; *N*: number.

**Table 2:** Anti-ZIKV antibody titer in blood donor samples after MNT tests.

ZIKV Antibody Titer	Number of Sample *N* (%)
15	11 (9.64)
22.5	28 (24.56)
45	21 (18.42)
67.5	18 (15.78)
135	14 (12.28)
202	1 (0.87)
202.5	3 (2.63)
405	7 (6.14)
607.5	6 (5.26)
1215	2 (1.75)
1837.5	2 (1.75)
3675	1 (0.87)
Total	114 (100)

**Table 3:** ZIKV seroprevalence according to the socio-demographic characteristics of the blood donors.

Variable	Positive *N* (%)	Odds Ratio IC95%	*p*-Value
Origin			0.0076 *
Ouagadougou	71 (27.73)	1	
Bobo-Dioulasso	43 (17.55)	0.55 [0.36–0.84]	
Gender			0.7034
Male	90 (23.25)	1	
Female	24 (21.05)	0.88 [0.53–1.46]	
Age			0.0148 *
18–24	27 (15.88)	1	
25–34	45 (22.84)	1.57 [0.93–2.66]	
35–44	31 (32.63)	2.57 [1.42–4.66]	
45–59	11 (28.20)	2.08 [0.93–4.67]	

* *p* < 0.05.

## Data Availability

Not applicable.

## References

[1] Sharma A., Lal S.K. (2017). Zika Virus: Transmission, Detection, Control, and Prevention. Front. Microbiol..

[2] Tinto B., Kania D., Samdapawindé Kagone T., Dicko A., Traore I., De Rekeneire N., Bicaba B.W., Hien H., Van de Perre P., Simonin Y. (2022). Dengue Virus Circulation in West Africa: An Emerging Public Health Issue. Med. Sci..

[3] Sow A., Loucoubar C., Diallo D., Faye O., Ndiaye Y., Senghor C.S., Dia A.T., Faye O., Weaver S.C., Diallo M. (2016). Concurrent Malaria and Arbovirus Infections in Kedougou, Southeastern Senegal. Malar. J..

[4] Marbán-Castro E., Goncé A., Fumadó V., Romero-Acevedo L., Bardají A. (2021). Zika Virus Infection in Pregnant Women and Their Children: A Review. Eur. J. Obstet. Gynecol. Reprod. Biol..

[5] Baud D., Gubler D.J., Schaub B., Lanteri M.C., Musso D. (2017). An Update on Zika Virus Infection. Lancet.

[6] Katzelnick L.C., Zambrana J.V., Elizondo D., Collado D., Garcia N., Arguello S., Mercado J.C., Miranda T., Ampie O., Mercado B.L. (2021). Dengue and Zika Virus Infections in Children Elicit Cross-Reactive Protective and Enhancing Antibodies That Persist Long Term. Sci. Transl. Med..

[7] Gubler D.J., Vasilakis N., Musso D. (2017). History and Emergence of Zika Virus. J. Infect. Dis..

[8] Duffy M.R., Chen T.-H., Hancock W.T., Powers A.M., Kool J.L., Lanciotti R.S., Pretrick M., Marfel M., Holzbauer S., Dubray C. (2009). Zika Virus Outbreak on Yap Island, Federated States of Micronesia. N. Engl. J. Med..

[9] Cao-Lormeau V.M., Roche C., Teissier A., Robin E., Berry A.L., Mallet H.P., Sall A.A., Musso D. (2014). Zika Virus, French Polynesia, South Pacific, 2013. Emerg. Infect. Dis..

[10] Brasil P., Calvet G.A., Siqueira A.M., Wakimoto M., de Sequeira P.C., Nobre A., de Souza Borges Quintana M., de Mendonça M.C.L., Lupi O., de Souza R.V. (2016). Zika Virus Outbreak in Rio de Janeiro, Brazil: Clinical Characterization, Epidemiological and Virological Aspects. PLoS Negl. Trop. Dis..

[11] Musso D., Ko A.I., Baud D. (2019). Zika Virus Infection—After the Pandemic. N. Engl. J. Med..

[12] Moore D.L., Causey O.R., Carey D.E., Reddy S., Cooke A.R., Akinkugbe F.M., David-West T.S., Kemp G.E. (1975). Arthropod-Borne Viral Infections of Man in Nigeria, 1964-1970. Ann. Trop. Med. Parasitol..

[13] Beltrán-Silva S.L., Chacón-Hernández S.S., Moreno-Palacios E., Pereyra-Molina J.Á. (2018). Clinical and Differential Diagnosis: Dengue, Chikungunya and Zika. Rev. Médica del Hosp. Gen. México.

[14] Muller D.A., Depelsenaire A.C.I., Young P.R. (2017). Clinical and Laboratory Diagnosis of Dengue Virus Infection. J. Infect. Dis..

[15] Mangada M.M., Rothman A.L. (2005). Altered Cytokine Responses of Dengue-Specific CD4 + T Cells to Heterologous Serotypes. J. Immunol..

[16] Katzelnick L.C., Narvaez C., Arguello S., Mercado B.L., Collado D., Ampie O., Elizondo D., Miranda T., Carillo F.B., Mercado J.C. (2020). Zika Virus Infection Enhances Future Risk of Severe Dengue Disease. Science.

[17] Letizia A.G., Pratt C.B., Wiley M.R., Fox A.T., Mosore M., Agbodzi B., Yeboah C., Kumordjie S., Di Paola N., Assana K.C. (2022). Retrospective Genomic Characterization of a 2017 Dengue Virus Outbreak, Burkina Faso. Emerg. Infect. Dis..

[18] Ridde V., Agier I., Bonnet E., Carabali M., Dabiré K.R., Fournet F., Ly A., Meda I.B., Parra B. (2016). Presence of Three Dengue Serotypes in Ouagadougou (Burkina Faso): Research and Public Health Implications. Infect. Dis. Poverty.

[19] Lim J.K., Seydou Y., Carabali M., Barro A., Dahourou D.L., Lee K.S., Nikiema T., Namkung S., Lee J.S., Shin M.Y. (2019). Clinical and Epidemiologic Characteristics Associated with Dengue during and Outside the 2016 Outbreak Identified in Health Facility-Based Surveillance in Ouagadougou, Burkina Faso. PLoS Negl. Trop. Dis..

[20] Jaenisch T., Tam D.T.H., Kieu N.T.T., Ngoc T., Nam N.T., Van Kinh N., Yacoub S., Chanpheaktra N., Kumar V., See L.L.C. (2016). Clinical Evaluation of Dengue and Identification of Risk Factors for Severe Disease: Protocol for a Multicentre Study in 8 Countries. BMC Infect. Dis..

[21] Franco L., di Caro A., Carletti F., Vapalahti O., Renaudat C., Zeller H., Tenorio A. (2010). Recent Expansion of Dengue Virus Serotype 3 in West Africa. Eurosurveillance.

[22] Amarasinghe A., Kuritsky J.N., William Letson G., Margolis H.S. (2011). Dengue Virus Infection in Africa. Emerg. Infect. Dis..

[23] Phoutrides E.K., Coulibaly M.B., George C.M., Sacko A., Traore S., Bessoff K., Wiley M.R., Kolivras K.N., Adelman Z., Traore M. (2011). Dengue Virus Seroprevalence among Febrile Patients in Bamako, Mali: Results of a 2006 Surveillance Study. Vector-Borne Zoonotic Dis..

[24] Onoja A.B., Adeniji J.A., Olaleye O.D. (2016). High Rate of Unrecognized Dengue Virus Infection in Parts of the Rainforest Region of Nigeria. Acta Trop..

[25] Fagbami A.H., Onoja A.B. (2018). Dengue Haemorrhagic Fever: An Emerging Disease in Nigeria, West Africa. J. Infect. Public Health.

[26] Sanou A.S., Dirlikov E., Sondo K.A., Kagoné T.S., Yameogo I., Sow H.E., Adjami A.G., Traore S.M., Dicko A., Tinto B. (2018). Building Laboratory-Based Arbovirus Sentinel Surveillance Capacity during an Ongoing Dengue Outbreak, Burkina Faso, 2017. Heal. Secur..

[27] Brès P. (1970). Données Récentes Apportées Par Les Enquêtes Sérologiques Sur La Prévalence Des Arbovirus En Afrique, Avec Référence Spéciale à La Fièvre Jaune. Bull. World Health Organ..

[28] Marchi S., Viviani S., Montomoli E., Tang Y., Boccuto A., Vicenti I., Zazzi M., Sow S., Diallo A., Idoko O.T. (2020). Zika Virus in West Africa: A Seroepidemiological Study between 2007 and 2012. Viruses.

[29] Diarra I., Nurtop E., Sangaré A.K., Sagara I., Pastorino B., Sacko S., Zeguimé A., Coulibaly D., Fofana B., Gallian P. (2020). Zika Virus Circulation in Mali. Emerg. Infect. Dis..

[30] Piantadosi A., Kanjilal S. (2020). Diagnostic Approach for Arboviral Infections in the United States. J. Clin. Microbiol..

[31] Simonin Y., Loustalot F., Desmetz C., Foulongne V., Constant O., Fournier-Wirth C., Leon F., Molès J.P., Goubaud A., Lemaitre J.M. (2016). Zika Virus Strains Potentially Display Different Infectious Profiles in Human Neural Cells. EBioMedicine.

[32] Tarnagda Z., Cissé A., Bicaba B.W., Diagbouga S., Sagna T., Ilboudo A.K., Tialla D., Lingani M., Sondo K.A., Yougbaré I. (2018). Dengue Fever in Burkina Faso, 2016. Emerg. Infect. Dis..

[33] Ritchie S.A., Cortis G., Paton C., Townsend M., Shroyer D., Zborowski P., Hall-Mendelin S., Van Den Hurk A.F. (2013). A Simple Non-Powered Passive Trap for the Collection of Mosquitoes for Arbovirus Surveillance. J. Med. Entomol..

[34] Meda N., Salinas S., Kagoné T., Simonin Y., Van de Perre P. (2016). Zika Virus Epidemic: Africa Should Not Be Neglected. Lancet.

[35] Weger-Lucarelli J., Rückert C., Chotiwan N., Nguyen C., Garcia Luna S.M., Fauver J.R., Foy B.D., Perera R., Black W.C., Kading R.C. (2016). Vector Competence of American Mosquitoes for Three Strains of Zika Virus. PLoS Negl. Trop. Dis..

[36] Fernandes R.S., O’connor O., Bersot M.I.L., Girault D., Dokunengo M.R., Pocquet N., Dupont-Rouzeyrol M., Lourenço-De-oliveira R. (2020). Vector Competence of Aedes Aegypti, Aedes Albopictus and Culex Quinquefasciatus from Brazil and New Caledonia for Three Zika Virus Lineages. Pathogens.

[37] Katzelnick L.C., Gresh L., Halloran M.E., Mercado J.C., Kuan G., Gordon A., Balmaseda A., Harris E. (2017). Antibody-Dependent Enhancement of Severe Dengue Disease in Humans. Science.

[38] HUANG Y.-M. (2004). The Subgenus Stegomyia of Aedes in the Afrotropical Region with Keys to the Species (Diptera: Culicidae). Zootaxa.

[39] Gillies M.T., Coetzee M. (1987). A Supplement to the Anophelinae of the South of the Sahara (Afrotropical Region). Publ. South African Inst. Med. Res..

[40] Gillies M.T., De Meillon B. (1968). The Anophelinae of Africa South of the Sahara (Ethiopian Zoogeographical Region). Publ. South African Inst. Med. Res..

[41] Faye O., Freire C.C.M., Iamarino A., Faye O., de Oliveira J.V.C., Diallo M., Zanotto P.M.A., Sall A.A. (2014). Molecular Evolution of Zika Virus during Its Emergence in the 20th Century. PLoS Negl. Trop. Dis..

[42] Raulino R., Thaurignac G., Butel C., Villabona-Arenas C.J., Foe T., Loul S., Ndimbo-Kumugo S.P., Mbala-Kingebeni P., Makiala-Mandanda S., Ahuka-Mundeke S. (2021). Multiplex Detection of Antibodies to Chikungunya, O’nyong-Nyong, Zika, Dengue, West Nile and Usutu Viruses in Diverse Non-Human Primate Species from Cameroon and the Democratic Republic of Congo. PLoS Negl. Trop. Dis..

[43] Liu S.Q., Li X., Deng C.L., Yuan Z.M., Zhang B. (2018). Development and Evaluation of One-Step Multiplex Real-Time RT-PCR Assay for Simultaneous Detection of Zika Virus and Chikungunya Virus. J. Med. Virol..

[44] Gray E.R., Heaney J., Ferns R.B., Sequeira P.C., Nastouli E., Garson J.A. (2019). Minor Groove Binder Modification of Widely Used TaqMan Hydrolysis Probe for Detection of Dengue Virus Reduces Risk of False-Negative Real-Time PCR Results for Serotype 4. J. Virol. Methods.

